# The Relationship between Fatty Acids and the Development, Course and Treatment of Rheumatoid Arthritis

**DOI:** 10.3390/nu14051030

**Published:** 2022-02-28

**Authors:** Wojciech Tański, Natalia Świątoniowska-Lonc, Mateusz Tabin, Beata Jankowska-Polańska

**Affiliations:** 1 Department of Internal Medicine, 4th Military Teaching Hospital, 50-981 Wroclaw, Poland; dr.wojciech.tanski@gmail.com; 2 Center for Research and Innovation, 4th Military Teaching Hospital, 50-981 Wroclaw, Poland; bpolanska@4wsk.pl; 3 Clinical Endocrinology Department, 4th Military Teaching Hospital, 50-981 Wroclaw, Poland; mateusz.tabin@gmail.com

**Keywords:** nutrition, fatty acids, rheumatoid arthritis

## Abstract

For this systematic review, a search of the relevant literature was conducted in the EMBASE and PubMed databases. We used the following terms: ‘rheumatoid arthritis’ in conjunction with ‘fatty acid’. The following inclusion criteria had to be satisfied for the studies to be included in the analysis: an RCT/observational/cohort study published in English. A total of seventy-one studies were analysed. The presented systematic review of the available data indicates that increased consumption of omega-3 fatty acids (FAs) may have a beneficial effect on human health by decreasing pain and disease activity in patients with RA. The beneficial effect of unsaturated FA on the clinical parameters of RA was demonstrated in all 71 studies analysed. The content of omega-3 FAs in the diet and the consumption of fish, which are their main source, may contribute to a reduced incidence of RA. FAs are an essential component in the synthesis of eicosanoids that exhibit anti-inflammatory properties. Due to the documented positive influence of unsaturated FAs on treatment outcomes, the use of a diet rich in long-chain unsaturated FAs should be the standard of care, along with pharmacotherapy, in the treatment of RA patients. An important element in the control of the treatment process should be the routine assessment of the quality of life of RA patients.

## 1. Introduction

Rheumatoid arthritis (RA) is one of the most common chronic autoimmune inflammatory diseases. The prevalence of RA in the world ranges from 0.5% to 1.0%, with the predominant percentage being women [1]. RA causes disability in 400,000–600,000 people. The first symptoms of the disease appear between the ages of 25 and 35. The highest incidence is observed in patients between 40 and 60 years of age. RA is a systemic disease of connective tissue characterised by nonspecific, symmetrical inflammation of mainly small and medium-sized joints, extraarticular lesions and systemic complications. It has periods of remissions and exacerbations and can lead to joint destruction, deformation, contractures and impaired function and, ultimately, is the most common cause of disability, progressive disability and premature death [2].

The causes of RA are not exactly known. Up to 60% of the risk for developing RA can be attributed to environmental susceptibility factors [3], but not many of them have been identified to date [4,5]. Several factors can influence the development of the disease. Some of them are disorders of the acquired immune response—they participate in the initiation and maintenance of disease development, with a special role of T lymphocytes recognising the body’s own antigens, e.g., citrullinated autoantigens, and supporting the production of autoantibodies with the same specificity, e.g., anti-CCP antibodies. The consequence of polyclonal B cell activation and excessive humoral immune response is the production of various autoantibodies, including RF, the presence of which is found in approximately 80% of RA patients. In such cases, the disease is characterised by a more severe clinical course. Additionally, a previous infection, especially viral, translates into the stimulation of the immune system in response to infection, which, in people predisposed to RA, may trigger an autoimmune reaction directed against the joint structures. Attention is drawn to the antigenic similarity of some bacteria or viruses and HLA-DRB1 and HLA-DRB4 histocompatibility antigens [6], often present in RA patients, which is conducive to the initiation of the autoimmune process. What is characteristic of RA is the appearance of pathological changes, which first occur in the joint lining. The B cells, macrophages and CD4+ helper T cells infiltrating the synovial stroma result in the spread of the synovium, which causes swelling and pain in the joints. In addition, the overproduction of inflammatory molecules, tumour necrosis factor (TNF), prostaglandin E2 (PGE2), interleukin (IL)-1 and cytokines induces chronic inflammation. TNF and IL-1 in particular play a significant role in the inflammatory process of joints in RA.

So far, researchers have focused mainly on the factors influencing the negative course of RA and the deterioration in the prognosis and treatment effectiveness, indicating smoking [7] and alcohol consumption [2,8] as the most important predictors. However, other modifiable risk factors for RA require further exploration. What is particularly interesting with regards to the nutritional factors is the consumption of unsaturated FAs. This is because of the role they play in the primary prevention of many chronic illnesses, including CVD diseases [9,10]. A few studies have shown that omega-3 FAs may be useful in treating some symptoms of RA [11,12], possibly through their anti-inflammatory effects [13]. For a long time, omega-3 FAs, especially eicosapentaenoic acid (EPA; 20: 5n-3) and docosahexaenoic acid (DHA; 22: 6n-3), have been regarded as factors with immunomodulating and anti-inflammatory properties [14]. Although it is widely believed that omega-6 FAs have a proinflammatory effect, some data indicate their immunomodulatory potential [15]. Therefore, the medium-chain omega-3 and omega-6 FAs ALA and LA, respectively, are essential nutrients for mammals, including humans. The relationship between polyunsaturated FA consumption and the risk of developing RA remains unclear, as the research results are inconclusive. Therefore, the aim of this systematic review is to summarise the available evidence from published epidemiological studies on the relationship between the consumption of products with high FA contents and the effectiveness of treatment in RA patients, as well as the role of FAs in the formation of RA.

## 2. Methods

For this systematic review, a search of the relevant literature was conducted in the EMBASE and PubMed databases. We used the following terms: ‘rheumatoid arthritis’ in conjunction with ‘fatty acid’. The references of the selected articles were analysed as well and included in the review. The following inclusion criteria had to be satisfied for the studies to be included in the analysis: an RCT/observational/cohort study published in English (Table 1). The present paper is in line with the guidelines for the preferred reporting items for systematic reviews and meta-analyses, i.e., the PRISMA statement [16].

**Figure 1:**
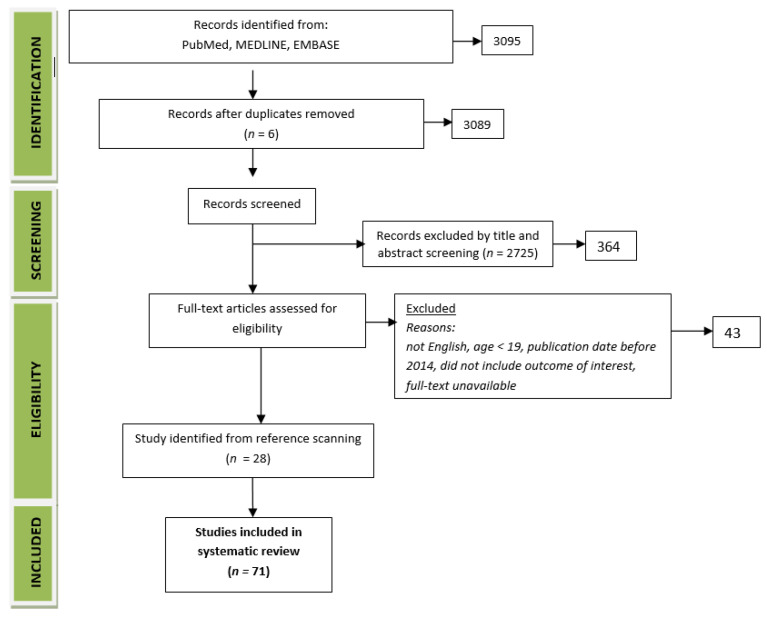
Study flow chart.

Human studies were included with clinical trials, observational studies and randomised controlled trials (RCT) (Figure 1). After excluding duplicates, 3089 articles were found. The papers were analysed and filtered for relevance. Studies were excluded if the results did not relate to the topic of the review or if their quality was evidently low. The final review included five articles and twelve referenced articles. Significant results or conclusions were extracted from each of these articles. The Bradford Hill [6] and critical appraisal skills program (CASP) criteria [88,89] were applied to assess the evidence for each topic. A total of seventy-one studies were analysed (33 randomised control trials, 10 clinical trials and 28 observational studies).

## 3. Classification of Fatty Acids

FAs are components of complex lipids, which are one of the basic macronutrients in the human diet. FAs constitute approximately 95% fat, and their composition influences the physiological role and physicochemical features [90]. From a physiological point of view, dietary fat primarily provides the energy necessary for the proper development and maintenance of vital functions. FAs are also involved in numerous metabolic processes, and their derivatives are active in many signalling pathways. The type of FAs contained in the diet, which often have different effects on the human body, determine the physiological and biochemical roles of dietary fat. Increased consumption of certain acids may promote health, which is mainly due to their proportion between saturated, monounsaturated and polyunsaturated FAs.

EPA and DHA, as well as omega-6 FAs, e.g., arachidonic acid (AA), are a component of cellular phospholipids. EPA is a precursor to prostaglandins and leukotrienes. NOmega-3 FAs supplementation can compete with AA for incorporation in membrane phospholipids. Furthermore, omega-3 FAs regulate proinflammatory and immune processes, competing for enzymes that convert them into eicosanoids, thus reducing the synthesis of inflammatory prostaglandins and leukotrienes. Omega-3 FAs play a role in numerous physiological processes involving membrane fluidity, receptor attraction, eicosanoid synthesis, gene expression, cell signalling and cytokine and pro-resolving mediator production.

**Figure 2:**
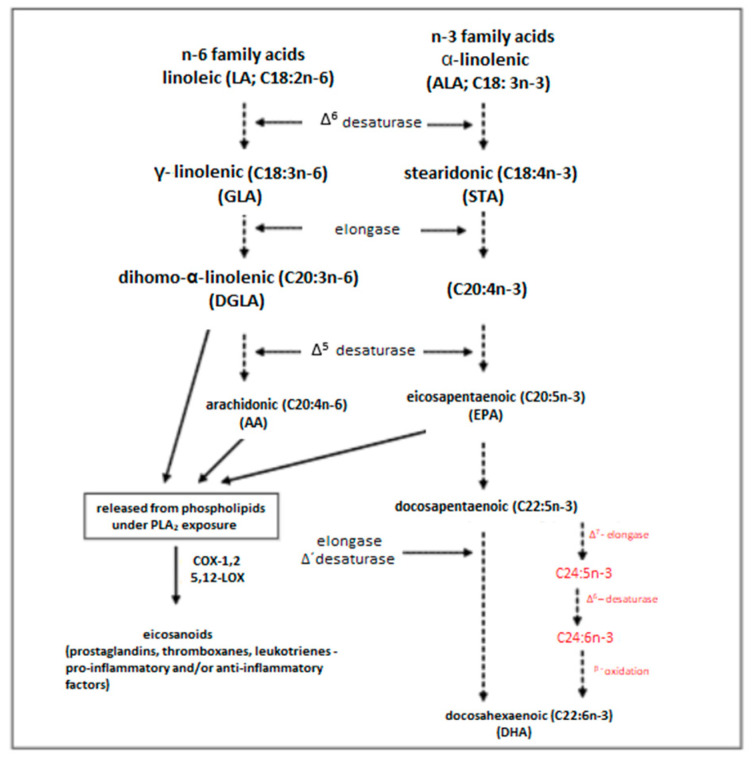
Synthesis of long-chain polyunsaturated acids from their precursors, i.e., linoleic acid (LA) and α-linolenic acid (ALA) [92].

The main nutritional form of omega-3 FA in most people is plant-derived alpha-linolenic acid (ALA; 18: 3n-3) (Figure 2) [91]. Following a number of consecutive desaturation and elongation steps, ALA is metabolised into long-chain EPA and DHA. This process directly competes with the desaturation and elongation of the much more abundant omega-6 FAs [92]. Phytoplankton has the ability to synthesise Omega-3 FAs, which are then transferred up through the food chain to fish via zooplankton. Due to the inability to synthesise ALA in humans, most of the EPA and DHA in the human body comes from the diet. Particularly good sources of these PUFAs are marine foods, e.g., deep sea fatty fish, or fish oil supplements. The parent FA of the n-6 family is linoleic acid (LA; 18: 2n-6). This omega-6 FA is more often obtained from the diet than any omega-3 FA. Its exceptionally good sources are vegetable oils, e.g., sunflower, soya bean and corn oils. The desaturation and elongation of LA result in the formation of longer omega-6 FA chains, including gamma-linolenic acid and arachidonic acid.

## 4. The Involvement of Fatty Acids in the Development of RA

Essential fatty acids released from phospholipids become precursors for the intrinsic synthesis of eicosanoids. These compounds include PG, prostacyclins (PGI), thromboxanes (TX) and leukotrienes. Eicosapentaenoic acid is converted to trienoic compounds (PGE3, PGI3, TXA3 and LTB5); linoleic acid is converted to monoenoic compounds (PGI1 and TXA1) and AA is a precursor to dienic compounds (PGE2, TXA2 and LTB4). Eicosanoids differ in their biological functions and, for this reason, often exhibit opposing actions. They influence, among others, the regulation of cardiovascular function, blood pressure, coagulation, plasma triglyceride levels, immune response and inflammatory processes [93]. The products of n-3 and n-6 EFA metabolism influence cellular biochemical processes; however, their different structures determine different activities; therefore, a properly balanced amount of both groups of acids in the diet is very important. Eicosanoids formed from AA already in small amounts show high biological activity and may exhibit prothrombotic and proinflammatory effects. On the other hand, eicosanoids, which are formed from n-3 EFAs, show anticoagulant, anti-inflammatory and vasodilatory effects [94,95]. An adequate supply of omega-3 fatty acids has a positive effect on reducing the production of highly proinflammatory eicosanoids (PG2, TX2 and LT4); increasing the production of weakly proinflammatory eicosanoids (PG3, TX4 and LT5); regulating the expression and activity of COX enzymes 5-LOX and the secretion of proinflammatory cytokines TNF, IL-1 and -6; allows a more effective control of granulocyte and macrophage activity; regulates adequate levels of resolvins and protease synthesis and helps to extinguish inflammation in the body [96].

The dietary consumption of omega-3 FAs may play an important role in the aetiology of RA. In their study, Kosińska et al. [70] found that polyunsaturated FAs constitute 13.5% of all FAs in the synovial fluid of patients with osteoporosis and RA, and the composition of the synovial fluid does not depend on the patient’s age. Similarly, a study by de Pablo et al. [63] revealed a significant relationship between the level of LA and preRA (OR 0.29; 95% CI 0.12–0.75) and the risk of developing RA. However, there was no association with other omega-3 or omega-6 FAs. In a study by Di Giuseppe et al. [71], dietary omega-3 FAs consumption exceeding 0.21 g/d was associated with a 35% decrease in the risk of developing RA ((RR) 0.65; 95% CI 0.48–0.90), as compared with a lower consumption of these fatty acids. However, long-term consumption greater than 0.21 g/d was associated with a 52% reduced risk of developing RA (95% CI 29–67%).

According to the available studies, polyunsaturated long-chain FAs may have prophylactic properties in the formation of RA. In their study, Gan et al. [68] assessed the relationship between the use of omega-3 FA supplements and the content of omega-3 FAs in RBC membranes, as well as the formation of anti-CCP autoantibodies in a population free of RA yet genetically predisposed to this disease. Positive anti-CCP2 antibodies were found in 30 patients, and 47 with negative autoantibody results qualified for the study. The likelihood of developing anti-CCP2 was inversely proportional to the total omega-3 FA content in the RBC (OR: 0.47; 95% CI: 0.24–0.92, for the s.d. increase), suggesting that omega-3 FAs may have a protective effect against developing RA in its preclinical stage.

A study conducted by Rodriguez-Carrio et al. [69] demonstrated lower levels of palmitoleic, palmitic, arachidonic, oleic, EPA and DHA acids in RA patients. These patients had an overrepresented NEFA profile, characterised by an increased content of stearic acid and a decreased content of EPA and DHA, as compared to healthy controls (*p* = 0.002). This was associated with clinical features (RF, erosions and shared epitope); increased expression of IFNγ in CD4+ T cells (*p* = 0.002) and serum environment enriched for Th1 (IFNγ, CXCL10 and CCL2, *p* < 0.005). These authors conducted in vitro tests that showed that an imbalance between FAs may be the cause of IFNγ production by CD4+ T cells. The clinical response to the blockade of TNF-α had an effect on the NEFA-level changes. Thus, the clinical features of the aggressive form of RA and an increased response of Th1 cells are associated with a changed NEFA profile in patients with RA.

The available studies have shown that supplementation with omega-3 FAs may be associated with the risk of developing RA [72,76,78,81,83]. In an observational study by Rosell et al. [76], the consumption of fatty fish was associated with a moderately reduced risk of developing rheumatoid arthritis (OR 0.8 (95% confidence interval = 0.6–1.0)). In the Pedersen et al. study [78], an increase in the intake of 30 g/d of fatty fish (≥8 g fat/100 g fish) was associated with a 49% reduction in the risk of developing RA (*p* = 0.06), whereas the intake of medium-fat fish (3–7 g fat/100 g fish) was associated with a significantly increased risk of developing RA. Additionally, the frequency of fish consumption had an effect on the development of RA. Shapiro et al. [83] demonstrated that the consumption of cooked or baked fish was associated with a reduced risk of rheumatoid arthritis, and this risk was significantly lower when >1 serving per week was consumed compared with 1 serving. Similarly, the consumption of olive oil or cooked vegetables significantly reduced the risk of RA (OR: 0.38 and 0.24, respectively) [81]. In the study by Lee and Park [72], the levels of ALA, EPA and omega-3 index (EPA + DHA) in erythrocytes were significantly lower in RA patients than in the controls. A regression analysis showed that the levels of ALA and EPA and the ratio of EPA to AA were negatively associated with RA risk. The PGE2 concentration was significantly decreased, with an increased DHA concentration in the erythrocytes of RA patients.

## 5. The Role of Fatty Acids in the Treatment of RA

A few studies have shown that omega-3 FAs may be useful in treating some of the RA symptoms [11,12], possibly through their anti-inflammatory effects [13]. For a long time, omega-3FAs, especially EPA (20: 5n-3) and DHA (22: 6n-3), have been regarded as factors with immunomodulating and anti-inflammatory properties [14]. In a study by Proudman et al. [20], the impact of unsaturated FA consumption (omega-3, eicosapentaenoic acid and docosahexaenoic acid) on the outcome of patients treated for RA was confirmed. The study included patients with RA lasting <12 months who were DMARD-naïve and randomised to a high-dose fish oil group (5.5 g/d) or a low-dose fish oil group (0.4 g/d for masking the purpose). The trial assessed the failure of a triple therapy with disease-modifying antirheumatic drugs (DMARD). The high-dose EFA group displayed a significantly lower failure of triple DMARD therapy (HR 95% CI 0.10–0.54; *p* = 0.0006) after adjusting for smoking history, baseline anti-CCP and shared epitope. This group of patients was also characterised by a significantly higher rate of remission according to ACR as compared to the control group (HRs = 2.09 (95% CI 1.02–4.30; *p* = 0.04) adjusted).

A study by Gan et al. [66] analysed the relationship between RF, anti-CCP2 Ab and the percentage of omega-3 FAs in RBC membranes, as well as the relationship between the reported use of omega-3 FA supplements and the incidence of anti-CCP2 Ab and RF. It was shown that there was an inverse association between the increase in omega-3 FA% in RBC and RF in participants who displayed shared epitope positivity (OR 0.27, 95% CI 0.10–0.79). No such association was observed in shared epitope negative participants. There were similar associations with anti-CCP positivity in SE-positive participants (OR 0.42, 95% CI 0.20–0.89). However, no such relationships were observed in SE-negative participants. In the SERA cohort, there was an association between the use of n-3 FA supplements and a lower incidence of RF positivity in SE-positive participants at the baseline (OR 0.32, 95% CI 0.12–0.82). There was no such relationship in shared epitope-negative participants. Similar trends were observed with anti-CCP2; however, they were not significant. Thus, n-3 FAs may have a potential protective influence on autoimmunity associated with RA, which is most evident in individuals who are genetically susceptible to RA in HLA class II.

Jeffery et al. [67] in their study showed that the concentration of PC EPA is associated with the clinical improvement of anti-TNF therapy in vivo and prevents the influence of ETN on Th17 cells in vitro. Thus, EPA supplementation may be an easy way to improve the outcomes of anti-TNF treatment in RA patients through Th17 frequency suppression. On the other hand, Beyer et al. [64] showed that there was an association between seafood consumption and a better outcome in RA treatment. These authors found a correlation between the omega-3 index >8, observed in 14% of patients, and higher VAS scores (*p* = 0.004) assessing the patient’s global health.

The effects of pharmacological treatment on the disease activity in RA may be complemented by including mussels in the patient’s diet. Such an addition can also contribute to the reduction of fatigue and pain in RA patients. In a study by Lindqvist [18], patients on a blue mussel diet had lower CRP, fewer tender joints, significantly improved global health and reduced pain and fatigue. In another study by Lindqvist [17], changes in the increase of omega-3 FAs EPA and DHA were observed in a group of patients who consumed blue mussels. In a study by Barebring et al. [65], a diet rich in fish, crustaceans, fruit and vegetables and whole grains was associated with a reduction in ESR (B = −2.4, *p* = 0.002) and hs-CRP (B = −0.6, *p* = 0.044). However, it was not associated with disease activity (DAS-28).

A study by Fu et al. [21] demonstrated a significant difference in the clinical disease activity index (CDAI) and disease activity scale (DAS28) after a 6-month intervention with the use of hard-shelled mussel lipid extract (Mytilus coruscus). Furthermore, there was a significant decrease in interleukin (IL)-1β, PGE2 and TNF-α but not IL-6 in this patient group and a significant increase in IL-10, indicating the potential of hard-shelled mussels as an adjunct to rheumatoid arthritis.

In another study [19], the patients with RA were divided into two food groups. In the first one, their food was enriched with Schizochytrium sp. microalgae oil (2.1 g DHA/d) and, in the second, with sunflower oil (placebo). The participants consumed the foods for 10 weeks (crossover). During this time, they maintained their regular intake of RA medications. The daily consumption of DHA reduced the sum of swollen and tender joints (66/68) from 13.9 ± 7.4 to 9.9 ± 7.0 (*p* = 0.010) and the total DAS28 index from 4.3 ± 1.0 to 3.9 ± 1.2 (*p* = 0.072). On the other hand, the consumption of sunflower oil (placebo) increased the content of LA and AA in EL (erythrocyte lipids), which mainly consist of erythrocyte membranes (*p* < 0.05). Patients supplemented with DHA presented a two-fold increase in the amount of DHA in the EL, and their AA/EPA and AA/DHA ratios were significantly reduced. These authors observed a significant reduction in the concentration of thromboxane B2 derived from AA and the ability of the blood to convert AA into 5-hydroxyeicosatetraenoic acid, which is a proinflammatory 5-lipoxygenase product. On the other hand, there was a significant increase in the levels of maresin/resolvin precursors derived from DHA and 14-/17-hydroxydocosahexaenoic acid as the result of DHA supplementation. Therefore, it can be concluded that DHA supplementation from microalgae reduces disease activity in RA patients, along with shifting the balance of lipid mediators derived from AA and DHA towards the anti-inflammatory/proliferative state.

In a study by Beyer et al. [60], the total concentration of FAs was higher in patients with active RA than in those in remission (*p* = 0.047). Similarly, RA patients treated with prednisolone had a higher total concentration of FAs as compared to those who did not receive prednisolone (*p* = 0.043). Although several single FAs varied with regards to the activity status of RA disease, prednisolone treatment or periodontal status, only C15:0 showed a positive association with CRP (*p* < 0.01, R = 0.30).

The Mustonem study [61] analysed the composition of infrapatellar fat pad (IFP) and synovial fluid (SF) from the knees of patients with RA and OA who had total joint replacement surgery. Joint diseases caused a significant decrease in the share of omega-6 FAs in the synovial fluid of OA and RA patients. The share of total MUFAs increased in SF in both RA and OA patients. As for IFP, the shares of 20: 4n-6, total omega-6 FA and 22: 6n-3 were lower in patients with RA. They also had a lower omega-3 FA product/precursor ratio compared to OA patients. The complex changes in FA signatures could contribute to the inflammatory processes and the destruction of cartilage in the knees of OA and RA patients, but they could also limit them. In contrast, in a study by Nasriati et al. [62], no correlation was found between FFA and the levels of TNF-α and the levels of VCAM-1 in RA patients. However, there was a negative correlation between the level of FFA and the level of VCAM-1 in RA patients.

Differences in the perception of clinical improvement after the introduction of dietary PUFAs may be due to the presence of specific genetic variants altering the ability of individuals to convert dietary MC-PUFAs to LC-PUFAs. In recent years, there has been a growing number of studies demonstrating population differences in the metabolic efficiency of the PUFA pathway due to genetic variants in fatty acid desaturase genes (FADS). Some studies have indicated that the FADS1 (Δ5-desaturase) step of PUFA biosynthesis appears to be the most genetically regulated step of PUFA biosynthesis in humans. However, most studies in the field have pointed to the FADS2 (Δ-6 desaturase) step as a critical step limiting the post-synthesis of LC-PUFAs such as AA, EPA and DHA [97,98]. Furthermore, different fatty acids in the diet (from heterogeneous diets) can affect several points in the biosynthetic pathway. Some PUFAs serve as enzymatic substrates for steps early in the pathway, whereas others serve as product inhibitors for the same enzymatic steps. High concentrations of LC-PUFAs, such as AA (derived from the conversion of LA to AA), may then affect the levels of proinflammatory eicosanoids, which, in turn, appear to be associated with elevated markers of low-level systemic inflammation, such as CRP, and increase the risk of diseases such as atherosclerosis [99,100]. To date, this hypothesis has been tested in heterogeneous human populations that also have high interindividual variability in the dietary concentrations of MC- and LC-PUFAs and in populations that are typically established by specific proinflammatory clinical conditions [101].

The supplementation of omega-3 FAs may support RA therapy. Das Gupta et al. [77], in their study, gave patients indomethacin (75 mg/d) or indomethacin (75 mg/d) and omega-3 FAs (3 g/d) over 12 weeks. Both groups showed moderate improvement in disease activity after 12 weeks of treatment. Physical functioning, physical role, body pain, general health, vitality, social functioning, grip strength and duration of morning stiffness improved significantly in the combination group compared with the indomethacin-only treatment group.

In the study by Aryaeian et al. [27], the group taking CLAs and vitamin E at the above doses had significantly lower ESR levels and significantly lower white blood cell counts compared to the placebo group. In addition, CLA supplementation reduces the SBP levels and mean arterial pressure and decreases the erythrocyte sedimentation rate of RA patients [29]. In the study by Ormseth et al. [74], the serum FFAs levels were associated with the HOMA-IR (*p* = 0.011), CRP (*p* = 0.01), triglycerides (*p* = 0.005) and Framingham risk scores (*p* = 0.048).

The use of an anti-inflammatory diet containing fish oil significantly reduces the number of tender and swollen joints and duration of morning stiffness of RA patients [24,30,34,35,36,37,40,41,43,45,46,47,48,49,52,57,59,73,75,77,79,84]. In a study by Adam O et al. [52], a fish oil diet resulted in greater EPA enrichment in erythrocyte lipids (244% vs. 217%) and less formation of leukotriene B (4) (34% vs. 8%, *p* > 0.01), 11-dehydro-thromboxane B (2) (15% vs. 10%, *p* < 0.05) and prostaglandin metabolites (21% vs. 16%, *p* < 0.003). In contrast, a diet low in AA alleviated the clinical signs of inflammation in RA patients and potentiated the beneficial effects of fish oil supplementation. Additionally, in a study by Kremer et al. [46], as a result of EPA and DHA supplementation, leukotriene B4 production by neutrophils decreased by 19 to 20% and interleukin-1 production by macrophages by 40.6–54.7% after 24 weeks. In a study conducted on 50 patients with RA, dietary supplementation with fish oil containing 60% omega-3 FAs resulted in a significant increase in the plasma EPA and monocyte lipid levels and clinical improvement in the study group [34]. In a study by Cleland et al. [57], after 12 weeks, the fish oil treatment group showed an improvement in the tender joint scores and grip strength, a reduction in the mean duration of morning stiffness, a reduction in pain and a 30% reduction in leukotriene B4 production by isolated neutrophils stimulated in vitro. Gruenwald et al. [79], in addition to a reduction in the duration of morning stiffness and a reduction in the number of painful and swollen joints at 6 and 12 weeks post-study, observed a 60% reduction in pain among patients taking EPA and DHA in the form of fish oil concentrate.

In the studies analysed, taking omega-3 FAs resulted in taking significantly less analgesic and antirheumatic preparations [30,38,39,43,44,51,58]. In the study by Lau et al. [39], this effect reached a maximum at month 12 and persisted until month 15. However, no change in the clinical and laboratory parameters of RA activity was observed in association with reduced NSAID consumption. Similarly, in a study by Brzeski et al. [44], patients taking GLA-rich evening primrose oil reduced the dose of NSAIDs and achieved clinical improvement. Additionally, GLA-rich borage seed oil significantly reduced the signs and symptoms of disease activity in patients with rheumatoid arthritis (*p* < 0.05) [40]. An overall clinical response (significant GLA administration) reduces joint inflammation in patients with rheumatoid arthritis by inhibiting IL-1 beta release from LPS-stimulated human monocytes [80]. GLA induces a protein that reduces the stability of pro-IL-1 beta mRNA. IL-1 beta is important for the host defence, but the enhancement mechanism may be excessive in genetically predisposed patients. The reduction of IL-1 beta autoinduction may therefore be protective in some patients with endotoxic shock and diseases characterised by chronic inflammation [50,80]. Belch et al. [58] observed significant improvement and reduction in NSAID use in groups using EPO and EPO with fish oil for 12 months. Moreover, the discontinuation of supplementation resulted in functional deterioration after 3 months in those receiving active treatment. Geusens et al. [38] found that patients taking 2.6 g/d of omega-3 FAs achieved significant improvements in patient global assessment and pain and reduction in antirheumatic medication. In the study by Galarraga et al. [30] of 49 patients, 19 (39%) in the cod liver oil group and five (10%) in the placebo group were able to reduce their daily NSAID requirements by >30%. There were no differences between the groups in the clinical parameters of RA disease activity or in the observed side effects.

In addition to the perceived subjective change in the clinical condition of RA patients after taking omega-3 FAs, researchers also observed changes at the biochemical level in the bodies of the patients studied [25,26,42,51,86,87]. In a study by Dawczynski et al. [26], in the group taking FAs (1.1 g a-linolenic acid, 0–7 g EPA and 0.1 g DPA and 0.4 g DHA and 50 mg/d AA), it was found that omega-3 FAs inhibited the immune response by significantly reducing the number of lymphocytes and monocytes. Omega-3FAs did not increase the oxidative stress biomarkers, such as 8-iso-PGF(2alpha) and 15-keto-dihydro PGF(2alpha), and DNA damage, such as 7,8-dihydro-8-oxo-2′-deoxyguanosine. In a study by Espersen et al. [42], the plasma interleukin-1 beta levels were significantly reduced in the study group after 12 weeks (*p* < 0.03) of taking 3.6 g/d omega-3 FAs. The anti-inflammatory effect of fish oil was also demonstrated in a study by Sperling et al. [86]. After fish oil supplementation, the AA:EPA ratio in neutrophil cell lipids decreased from 81:1 to 2.7:1, and the mean leukotriene B4 production decreased by 33%. There was also a 37% decrease in platelet-activating factor production at week 6. In a study by Cleland et al. [51], after 3 years of fish oil use, AA was 30% lower in the platelets and 40% lower in peripheral blood mononuclear cells in subjects taking fish oil. Serum thromboxane B2 was 35% lower, and whole-blood PGE2 stimulated by lipopolysaccharide was 41% lower with fish oil consumption compared with no fish oil. In a study by Kolahi et al. [25], in the fish oil supplementation group (1 g/d), the osteoprotegerin levels increased, while sRANKL, TNF-alpha and the sRANKL/osteoprotegerin ratio decreased, and there was a significant positive correlation between the sRANKL/osteoprotegerin ratio and TNF-alpha levels (r = 0.327, *p* = 0.040). The literature data suggest the involvement of the potent chemotactic factors 5-HETE and leukotriene B4 in inflammatory disease in humans [87]. A study of synovial fluid from patients with RA, spondyloarthropathy (SA) or noninflammatory arthropathy (NIA) showed that 5(S),12(R)-dihydroxy-6,8,10-(trans/trans/cis)-14-cis-eicosatetraenoic acid (leukotriene B4) in synovial fluid was significantly elevated in patients with RA and the rheumatoid factor present (*p* < 0.05, *n* = 14) and in patients with SA (*p* < 0.05, *n* = 10) compared with those with NIA (*n* = 9) [87]. The content of 5(S)-hydroxy-6,8,11,14-eicosatetraenoic acid (5-HETE), but not leukotriene B4, was significantly elevated in the synovial tissue of seven RA patients compared with four NIA subjects (*p* < 0.05). A single intraarticular corticosteroid injection significantly decreased the leukotriene B4 levels in the synovial fluid of six RA patients [87]. In the study by Bae et al. [28], there were no significant differences in the proinflammatory cytokines, CRP levels and disease severity in the groups taking quercetin with vitamin C (166 mg + 133 mg/capsule) and alpha-lipoic acid (300 mg/capsule). In the study by Dawczynski et al. [23], following the administration of 3 g/d omega-3 FAs, the AA/EPA ratio decreased from 6.5 ± 3.7 to 2.7 ± 2.1 in the plasma lipids and from 25.1 ± 10.1 to 7.2 ± 4.7 in the erythrocyte membranes (*p* ≤ 0.001). In the group taking GLA and in the group taking omega-3 FAs and GLA simultaneously, there was a strong increase in the GLA and dihomo-γ-linolenic acid concentrations in the plasma lipids, cholesterol esters and erythrocyte membranes. Jäntti et al. [56] showed that the decrease in EPA and increase in AA serum concentrations induced by evening primrose oil may not be beneficial in patients with rheumatoid arthritis in light of the role of these FAs as eicosanoid precursors. Decreases in essential FAs are associated with increased desaturase/elongation enzyme activity, increased eicosanoid production or metabolic changes secondary to a cytokine-mediated inflammatory response [85]. In a study by Fraser et al. [82] evaluating how changes in FFAs after a 7-day fast in rheumatoid arthritis (RA) patients will inhibit T-lymphocyte proliferation in vitro, it was demonstrated that both the concentration of the FFA mixture and the ratio of unsaturated and saturated fatty acids significantly affected lymphocyte proliferation in vitro (*p* < 0.0001).

The three studies reviewed did not show an association between omega-3 FAs intake and subjective clinical improvement [32,33,54]. In the study by Remans et al. [32], patients in the study group supplementing EPA, DHA, GLA and micronutrients showed a significant increase in the plasma levels of vitamin E (*p* = 0.015) and EPA, DHA and docosapentaenoic acid, with a decrease in the AA levels (*p* = 0.01). Similarly, in the study by Sundrarjun et al. [33], patients consuming foods low in omega-6 FAs and supplemented with omega-3 FAs at week 18 had significantly decreased linoleic acid, CRP and sTNF-R p55 concentrations and significant increases in EPA and DHA compared to the placebo group. At week 24, there was a significant reduction in the interleukin-6 and TNF-alpha levels in the group; however, no association with clinical improvement in the patients was observed. In the study by Haugen et al. [54], the 20: 3n-6 and 20: 4n-6 fatty acid concentrations were significantly reduced after 3.5 months on a vegan diet (*p* < 0.0001 and *p* < 0.01, respectively), but the concentrations increased to the baseline values with a lactovegetarian diet. The 20: 5n-3 concentration was significantly reduced after a vegan diet (*p* < 0.0001) and a lactovegetarian diet (*p* < 0.01).

## 6. Fatty Acids and the Quality of Life

One of the additional objectives of the present study was to analyse the association between FAs and the quality of life (QoL) of RA patients. Unfortunately, the literature does not present such studies with regards to RA. However, in recent years, two studies involving rheumatic patients have been published—these were patients suffering from systemic lupus erythematosus (SLE) and osteoporosis.

The available studies have shown insufficient levels of many energy-producing substrates, including physiological antioxidants, and a reduced content of omega-3 FAs in SLE patients. It has been demonstrated [101] that supplementation with omega-3 FAs in SLE increases the level of antioxidants and improves the functional efficiency and, thus, QoL in SLE patients [101]. A study by Arriens et al. [101] confirmed the positive effect of consuming omega-3 FAs on QoL in patients with SLE. In SLE patients taking omega-3, both the overall QoL measured with the SF-36 questionnaire and the Vitality/Fatigue subscale score statistically improved (respectively, 32.60 vs. 42.19, *p* = 0.0076; 29.44 vs. 39.17, *p* = 0.023). The researchers did not find any significant changes in the QoL among patients taking a placebo (olive oil).

The positive influence on the physical aspect of the QoL was confirmed among patients with OA. In a study by Kraemer et al. [102], the effect of topically applied cream that consisted of conjugated fatty acids (CFA) on the functional performance of patients diagnosed with OA of one or both knees was investigated. The physical aspect of the quality of life was assessed, and the tests included the knee range of motion (ROM), a medial step-down test, the unilateral anterior reach and timed ‘up-and-go’ from a chair and stair climbing. Shortening of the timed ‘up-and-go’ from a chair and improvement in stair climbing and the medial step-down test and an increase in ROM were observed in patients using the CFA cream.

## 7. Conclusions and Summary

The aetiology of RA is multifactorial. The disease is influenced by environmental factors, including diet. The content of omega-3 FAs in the diet and the consumption of fish, which are their main source, may contribute to a reduced incidence of RA. These FAs are an essential component in the synthesis of eicosanoids, which exhibit anti-inflammatory properties.

The presented systematic review of the available data indicates that the increased consumption of omega-3 FAs may have a beneficial effect on health by reducing the pain and disease activity in RA patients. The beneficial influence of unsaturated fatty acids on the clinical parameters of RA was demonstrated in all 71 analysed studies.

Due to the documented positive effect of unsaturated FAs on the results of treatment, the use of a diet rich in long-chain unsaturated FAs should be the standard of care, along with pharmacotherapy, in treating patients with RA. An important element in the control of the treatment process should be the routine assessment of the quality of life in RA patients.

## Figures and Tables

**Table 1:** Description of the included studies.

Lp.	Author Year	Study Design	Study Group	Intervention	Results
1	Lindqvist HM et al., 2019 [17]	RCT	39 RA women aged 25–65 years	CG: habitual diet (*n* = 19). IG: replacement of one meal per day, with a meal containing 75 g blue mussels or 75 g meat (*n* = 20). Duration 11 weeks, change between groups after 8 weeks of elimination.	GI patients differed in erythrocyte fatty acid profile compared to CG, with changes in the increase of omega-3 fatty acids: EPA and DHA at the group level. The fatty acid profile in plasma phospholipids and serum 1H NMR metabolites was not significantly different between diets. The change in the pattern of fatty acids in erythrocytes may be associated with a reduction in disease activity, although it cannot be excluded that factors other than omega-3 fatty acids potentiate this effect.
2	Lindqvist HM et al., 2018 [18]	RCT	39 RA women aged 25–65 years	CG: habitual diet (*n* = 19). IG: replacement of one meal per day, with a meal containing 75 g blue mussels or 75 g meat (*n* = 20). Duration 11 weeks, change between groups after 8 weeks of elimination.	A reduction in DAS28-CRP (*p* = 0.048) but not DAS28 was observed in the IG group. Blue mussel consumption was associated with moderate to good response to EULAR criteria and a reduction in RA symptoms. Blood lipid levels were unchanged.
3	Dawczynski C et al., 2018 [19]	RCT	38 RA patients aged 59.5 ± 12.4 years	IG: (*n* = 19) enriched with oil from the microalgae Schizochytrium sp. (2.1 g DHA/d) CG: (*n* = 19) with sunflower oil (placebo) Time: 10 weeks	In IG, daily DHA consumption led to a decrease in the sum of tender and swollen joints from 13.9 ± 7.4 to 9.9 ± 7.0 and the total DAS28 score from 4.3 ± 1.0 to 3.9 ± 1.2 in contrast to CG. An increase in LA and AA content in erythrocyte lipids was observed in the placebo group. In contrast, in the IG group the amount of DHA was doubled in EL, and the ratio of AA/EPA and AA/DHA decreased significantly.
4	Proudman SM et al., 2015 [20]	RCT	IG: 86 RA patients aged 56.1 ± 15.9 CG: 56 RA patients aged 55.5 ± 14.1	IG: 5.5 g/d omega-3 FAs + EPA + DHA CG: 0.4 g/d omega-3 FAs + EPA + DHA	IG patients had lower DMARD triple therapy failure rate (HR = 0.28) (95% CI 0.12–0.63; *p* = 0.002), higher ACR first remission rate (HRs = 2.17) (95% CI 1.07–4.42; *p* = 0.03) compared to CG. No differences in DAS28 and mHAQ or adverse events between IG and CG.
5	Fu Y et al., 2015 [21]	RCT	50 RA patients aged 28–75 years	IG: lipid extract from hard-shelled mussel (*Mytilus coruscus*) (HMLE) CG: Placebo Time: 6 months	The HMLE group showed significant improvement in DAS-28 disease activity score, clinical disease activity index (CDAI), decrease in TNF-α (tumour necrosis factor α), interleukin (IL)-1β and PGE2 (prostaglandin E2) after 6-month intervention. IL-10 was increased in both groups, significantly more in the HMLE group.
6	Park Y et al., 2013 [22]	RCT	IG: 41 RA patients aged 49.24 ± 10.46 CG: 40 RA patients aged 47.63 ± 8.78	IG: 2.09 g EPA and 1.165 g DHA CG: sunflower oil with high oleic acid content. Time: 16 weeks	The IG group showed a significant increase in erythrocyte levels of omega-3 FAs and EPAs and a decrease in omega-6 FAs, 18: 2n6, 20: 4n6 and 18: 1n9 compared with the placebo group. Supplementation with n-3 PUFAs had no significant effect on the need for non-steroidal anti-inflammatory drugs (NSAIDs), clinical symptoms of RA, or levels of cytokines, eicosanoids and bone turnover markers. In contrast, n-3 PUFA supplementation significantly reduced NSAID requirements and leukotriene B4 levels in patients who weighed more than 55 kg.
7	Dawczynski C et al., 2011 [23]	RCT	54 RA patients and 6 patients with psoriatic arthritis in mean age 56 ± 13 years	I: 3.0 g omega-3 FAs/d; II: 3.2 g GLA/d III: 1.6 g omega-3 FAs + 1.8 g GLA/d; IV: 3.0 g olive oil Time: 12 weeks.	In group I, the AA/EPA ratio decreased from 6.5 ± 3.7 to 2.7 ± 2.1 in plasma lipids and from 25.1 ± 10.1 to 7.2 ± 4.7 in erythrocyte membranes (*p* ≤ 0.001). A strong increase in GLA and dihomo-γ-linolenic acid was observed in plasma lipids, cholesterol esters and erythrocyte membranes in groups II and III.
8	Bahadori B et al., 2010 [24]	RCT	23 patients with moderate to severe RA	IG: 0.2 g fish oil emulsion/kg intravenously for 14 days, then 0.05 g fish oil/kg CG: 0.9% saline (placebo) intravenously for 14 days, then paraffin (placebo) taken orally as capsules.	The number of swollen joints was significantly lower in the omega-3 FA group compared with the placebo group after 1 week of infusion (*p* = 0.002) as well as after 2 weeks of infusion (*p* = 0.046). The number of tender joints also tended to be lower in the omega-3 FA group, although this did not reach statistical significance. Both the number of swollen and tender joints were significantly lower in the omega-3 FA group compared with the placebo group during and at the end of oral treatment.
9	Kolahi S et al., 2010 [25]	RCT	I: 40 RA female patients aged 50 (18–74) II: 43 RA female patients aged 50 (19–74)	I: fish oil 1 g/d II: no fish oil, conventional drugs Time: 3 months	In the fish oil supplementation group, osteoprotegerin levels increased, while sRANKL, TNF-alpha and the sRANKL/osteoprotegerin ratio decreased and there was a significant positive correlation between the sRANKL/osteoprotegerin ratio and TNF-alpha levels (r = 0.327, *p* = 0.040).
10	Dawczynski C et al., 2009 [26]	RCT	39 RA patients aged 57.9 ± 10.8 years	IG: 40 g fat in the form of 200 g yogurt with 3–8% fat, 30 g cheese with about 50% fat in dry matter, and 20–30 g butter daily; 1.1 g a-linolenic acid, 0–7 g EPA, 0.1 g DPA and 0.4 g DHA. 50 mg/d AA CG: dairy products with comparable fat content, 70 mg/d AA Time: 12 weeks and an 8-week elimination phase in between.	In the IG group, we found that omega-3 FAs inhibited the immune response by significantly reducing the number of lymphocytes and monocytes. N-3 LC-PUFAs did not increase oxidative stress biomarkers, such as 8-iso-PGF(2alpha) and 15-keto-dihydro PGF(2alpha), and DNA damage, such as 7,8-dihydro-8-oxo-2′-deoxyguanosine.
11	Aryaeian N et al., 2009 [27]	RCT	Gr. P: 22 RA patients aged 47.95 ± 11.14 Gr. C: 22 RA patients aged 46.23 ± 13.07 Gr. E: 21 RA patients aged 49.33 ± 11.89 Gr. CE: 22 RA patients aged 43.77 ± 12.75	C: CLAs 2.5 g equivalent to 2 g of a 50/50 mixture of cis 9-trans11 and trans 10-cis12 CLAs E: vitamin E 400 mg CE: CLAs and vitamin E at the above doses P: placebo. Time: 3 months	DAS28, pain and morning stiffness were significantly decreased in the Ci CE group compared with the P group (*p* < 0.05). Compared with baseline, ESR levels decreased significantly in groups C (*p* < or =0.05), E (*p* < or =0.05) and CE (*p* < or =0.001). The CE group had significantly lower ESR levels than the P group (*p* < 0.05) and a significantly lower white blood cell count compared with the other groups (*p* < 0.05).
12	Bae SC. et al., 2009 [28]	RCT	20 RA patients with the mean age of 52.1 ± 10.3 years	I: quercetin + vitamin C (166 mg + 133 mg/capsule) II: alpha-lipoic acid (300 mg/capsule) III: placebo for 4 weeks (3 capsules/day). Time: 4 weeks with a 2-week break before starting the next supplementation.	There were no significant differences in serum levels of proinflammatory cytokines and CRP between the study groups. Disease severity scale scores were not significantly different between study groups, although quercetin supplementation tended to reduce the VAS.
13	Aryaeian N et al., 2008 [29]	RCT	Gr. P: 22 RA patients aged 47.95 ± 11.14 Gr. C: 22 RA patients aged 46.23 ± 13.07 Gr. E: 21 RA patients aged 49.33 ± 11.89 Gr. CE: 22 RA patients aged 43.77 ± 12.75	C: CLAs 2.5 g equivalent to 2 g of a 50/50 mixture of cis 9-trans11 and trans 10-cis12 CLAs E: vitamin E 400 mg CE: CLAs and vitamin E at the above doses P: placebo. Time: 3 months	After supplementation, SBP levels decreased significantly in group C compared with groups E and P, and mean arterial pressure decreased significantly in groups C and CE. There were no significant differences in PGE2, triglycerides, cholesterol, LDL-C, HDL-C, LDL/HDL, cholesterol/HDL, fasting blood sugar, CRP, arylesterase activity, and platelet count between groups. Erythrocyte sedimentation rate decreased in C, E and CE groups.
14	Galarraga B et al., 2008 [30]	RCT	IG: 49 RA patients in median age 58 years CG: 48 RA patients in median age 61 years	IG: 10 g cod liver oil containing 2.2 g omega-3 Fas CG: air-filled placebo capsules. Time: 9 months	Of the 49 patients, 19 (39%) in the cod liver oil group and 5 (10%) in the placebo group were able to reduce daily NSAID requirements by >30%. There were no differences between the groups in clinical parameters of RA disease activity or in observed side effects.
15	Berbert AA et al., 2005 [31]	RCT	43 RA patients in mean age 49 ± 19 years	CG: soybean oil (placebo) IG1: 3 g/d omega-3 FAs from fish oil IG2: 3 g/d omega-3 FAs from fish oil + 9.6 mL olive oil. Time: 24 weeks	There was statistically significant improvement (*p* < 0.05) in IG1 and IG2 groups relative to CG in joint pain intensity, right and left hand grip strength at 12 and 24 w, duration of morning stiffness, onset of fatigue, Ritchie joint index for painful joints at 24 w, ability to bend to pick up clothing from the floor, and getting in and out of a car at 24 w. IG2 vs. CG showed additional improvement for duration of morning stiffness after 12 w, global patient assessment at 12 and 24 w, and from rheumatoid factor at 24 w. In addition, IG2 showed significant improvement in global patient assessment relative to IG1 at 12 w.
16	Remans PH et al., 2004 [32]	RCT	IG: 26 RA patients aged 52.97 ± 11.2 CG: 29 RA patients aged 59.57 ± 11.0	IG: 1.4 g EPA, 0.211 g DHA, 0.5 g-GLA and micronutrients CG: placebo Time: 4 months	There was no significant change in the number of tender joints or other clinical parameters in any of the study groups compared with baseline. In patients receiving nutrient supplementation but not placebo, there was a significant increase in plasma levels of vitamin E (*p* = 0.015) and EPA, DHA, and docosapentaenoic acid with a decrease in AA levels (*p* = 0.01).
17	Sundrarjun T et al., 2004 [33]	RCT	I: 23 RA patients aged 46.2 ± 0.5 II: 23 RA patients aged 46.0 ± 0.5 III: 14 RA patients aged 48.6 ± 0.7	I: low omega-6 FA diet + omega-3 FA supplement (fish oil), II: low omega-6 FA diet (placebo group) III: no special diet or intervention (control group).	At week 18, group I had significant decreases in linoleic acid, CRP, and sTNF-R p55 and significant increases in EPA and DHA compared with group III. There were no significant differences in clinical variables among the three groups. At week 24, there was a significant reduction in interleukin-6 and TNF-alpha in groups I and III.
18	Volker D et al., 2000 [34]	RCT	50 RA patients	IG: fish oil containing 60% omega-3 FAs supplemented at 40 mg/kg body weight/d. CG: diet naturally low in omega-6 FAs Time: 15 weeks	Dietary supplementation resulted in a significant increase in plasma EPA and monocyte lipid levels and clinical improvement in IG.
19	Sarzi-Puttini P et al., 2000 [35]	RCT	I: 25 RA patients aged 49.56 (32–64) II: 25 RA patients aged 50.28 (29–70)	IG: A diet high in unsaturated fats and low in saturated fats with hypoallergenic foods CG: control diet, well balanced Time: 24 weeks	Significant reductions in Ritchie index, number of tender and swollen joints, and ESR were obtained in IG.
20	Zurier RB et al., 1996 [36]	RCT	56 RA patients	IG: 2.8 gm/d GLA CG: placebo (sunflower oil capsules) Time: 6 months This was followed by a 6-month single-blind study during which all patients received GLA.	There was a statistically and clinically significant reduction in signs and symptoms of disease activity in RA patients in the IG group. During the second 6 months, improvements in disease activity were observed in both groups.
21	Leventhal LJ et al., 1994 [37]	RCT	RA patients	Black currant seed oil (BCSO) administered for 24 weeks	BCSO treatment reduced signs and symptoms of disease activity (*p* < 0.05). The overall clinical response was not better in the treatment group than in the placebo group.
22	Geusens P et al., 1994 [38]	RCT	I: 20 RA patients aged 56.2 ± 2 years II: 21 RA patients aged 57.2 ± 2 years III: 19 RA patients aged 59.2 ± 2 years	I: 6 capsules containing 1 g each of olive oil (placebo), II: 3 capsules each containing 1 g fish oil (1.3 g/d omega-3 FAs) plus 3 placebo capsules, III: 6 capsules containing 1 g each of fish oil (2.6 g/d omega-3 FAs) Time: 12 months	Patients taking 2.6 g/d of omega-3 FAs had significant improvements in patient global assessment, pain, and reduction in antirheumatic medication.
23	Lau CS et al., 1993 [39]	RCT	64 RA patients	I: 10 capsules of Maxepa (171 mg EPA+ 114 mg DHA) II: placebo Duration: 12 months, then 3 months placebo.	Patients taking Maxepa consumed significantly less NSAIDs compared with placebo from month 3 [71.1 (55.9–86.2)% and 89.7 (73.7–105.7)%]. This effect reached a maximum at month 12 and persisted until month 15. No change in clinical or laboratory parameters of RA activity was observed in association with reduced NSAID consumption.
24	Leventhal LJ et al., 1993 [40]	RCT	IG: 19 RA patients aged 58 ± 13 CG: 18 RA patients aged 50 ± 16	IG: 1.4 g/d GLA in borage seed oil CG: cottonseed oils (placebo).	Signs and symptoms of disease activity in patients with rheumatoid arthritis decreased significantly in the IG group (*p* < 0.05). The overall clinical response (significant change in four measurements) was also better in the treatment group (*p* < 0.05).
25	Kjeldsen-Kragh J et al., 1992 [41]	RCT	67 RA patients	I: corn oil (placebo), 7 g/day for 16 weeks, and naproxen, 750 mg/day for 10 weeks, followed by a gradual dose reduction to 0 mg/day over the next 3 weeks; II: 3.8 g EPA + 2.0 g DHA and naproxen, 750 mg/day for 16 weeks III: 3.8 g EPA + 2.0 g DHA and naproxen 750 mg/day for 10 weeks	Group II showed improvement in the duration of morning stiffness and global health score. In group III, for the duration of morning stiffness, the deterioration was significantly less compared to group I.
26	Espersen GT et al., 1992 [42]	RCT	32 RA patients	I: dietary supplementation with omega-3 FAs (3.6 g/d) II: placebo Time: 12 weeks	Plasma Interleukin-1 beta levels were significantly reduced in the study group after 12 weeks (*p* < 0.03). The clinical status of the patients improved in the group receiving fish oil (*p* < 0.02). It was concluded that dietary supplementation with n-3 fatty acids leads to a significant reduction in plasma IL-1 beta levels in patients with rheumatoid arthritis.
27	Nielsen GL et al., 1992 [43]	RCT	57 RA patients aged 61 (33–78) years IG: 29 patients CG: 28 patients	IG: 6 capsules of omega-3 FAs (3.6 g) CG: 6 capsules with a fat composition like the average Danish diet.	Significant improvement in morning stiffness and joint tenderness in the study group.
28	Brzeski M et al., 1991 [44]	RCT	40 RA patients with upper gastrointestinal lesions due to use of non-steroidal anti-inflammatory drugs I: 19 RA patients II: 21 RA patients	I: 540 mg/d GLA (evening primrose oil 6 g/d), II: placebo (olive oil 6 g/d).	Three Patients in each group reduced their NSAID dose. The GLA treatment group had a significant reduction in morning stiffness after 3 months of supplementation, and the placebo group had a reduction in pain and joint index after 6 months.
29	van der Tempel H et al., 1990 [45]	RCT	16 RA patients in mean age 53 years	IG: fish oil CG: placebo (fractionated coconut oil) Duration: 12 weeks	Joint swelling index and duration of morning stiffness were lower in IG than in CG. The relative amounts of EPA and DHA in plasma cholesterol ester and neutrophil membrane phospholipid fractions increased in the IG group, mainly at the expense of omega-6 Fas, and the mean in vitro production of leukotriene B4 by neutrophils decreased after 12 weeks of supplementation. Production of leukotriene B5 increased to significant amounts during fish oil treatment.
30	Kremer JM et al., 1990 [46]	RCT	49 RA patients I: 20 RA patients aged 59 (32–81) II: 17 RA patients aged 58 (30–80 III: 12 RA patients aged 58 (22–81)	I: dietary supplement with omega-3 FA-s (27 mg/kg EPA and 18 mg/kg DHA) daily II: 54 mg/kg EPA and 36 mg/kg DHA daily III: olive oil capsules containing 6.8 gm of oleic acid daily. Time: 24 weeks	Significant improvement from baseline in the number of tender and swollen joints was observed in groups I and II. A total of 5 of 45 clinical measurements were significantly changed from baseline in group III, 8 of 45 in group I, and 21 of 45 in group II during the study. Leukotriene B4 production by neutrophils decreased by 19% in group I and 20% in group II, whereas interleukin-1 production by macrophages decreased significantly by 40.6% in group I (*p* = 0.06) and 54.7% in group II (*p* = 0.0005).
31	Tulleken JE et al., 1990 [47]	RCT	I: 14 RA patients aged 52 (29–66) years II: 14 RA patients aged 58 (43–68) years	I: fish oil II: coconut oil enriched with alpha-tocopherol (placebo) Time: 3 months	The results of the study provide evidence that the beneficial effects of fish oil supplementation cannot be attributed to the antioxidant properties of alpha-tocopherol per se.
32	Magaro M et al., 1988 [48]	RCT	I: 6 RA female patients aged 37 (20–55) years II: 6 RA female patients 36 (20–50) years	I: diet high in PUFA supplemented with EPA and DHA II: diet high in saturated fatty acids.	Fish oil consumption resulted in subjective relief of symptoms of active rheumatoid arthritis and decreased neutrophil chemiluminescence.
33	Kremer JM et al., 1985 [49]	RCT	IG: 17 RA patients in mean age 55.2 years CG: 20 RA patients in mean age 56.5 years	IG: diet high in polyunsaturated fat and low in saturated fat, with daily supplementation (1.8 g) of EPA. CG: diet with lower ratio of polyunsaturated to saturated fats and placebo supplementation. Time: 12 weeks	At week 12 of the study, a reduction in morning stiffness time and number of tender joints was observed in the IG group. After discontinuation of the diet, there was a significant deterioration in the experimental group’s global assessment of disease activity, pain score, and number of tender joints.
34	Leeb BF et al., 2006 [50]	Clinical Trial	34 RA patients aged 61 ± 4.2 years	2 mL/kg (0.1 to 0.2 g fish oil/kg) of fish oil emulsion intravenously for 7 consecutive days Time: 5 weeks	56% achieved a DAS28 reduction > 0.6 in V2 (mean 1.52); 27% > 1.2. In V3, 41% of patients showed a DAS28 reduction > 0.6 (mean 1.06) and 36% > 1.2.
35	Cleland LG et al., 2006 [51]	Clinical Trial	I: 13 RA patients aged 51.1 ± 15.9 II: 18 RA patients 61.8 ± 9.9	I: no fish oil II: fish oil to provide 4–4.5 g EPA plus DHA daily or equivalent fish oil capsule dose (7 × 1 g capsules twice daily). Time: 3 years	After 3 years, AA was 30% lower in platelets and 40% lower in peripheral blood mononuclear cells in those taking fish oil. Serum thromboxane B2 was 35% lower and PGE2 in whole blood stimulated by lipopolysaccharide was 41% lower with fish oil consumption compared with no fish oil. NSAID use was reduced by 75% from baseline with fish oil intake (*p* < 0.05) and by 37% without fish oil (NS). Remission at 3 years was more common with fish oil use (72%) compared to no fish oil (31%).
36	Adam O et al., 2003 [52]	Clinical Trial	68 RA patients CG: west diet (*n* = 34) IG: anti-inflammatory diet with a daily intake of AA < 90 mg/d (*n* = 34)	CG: placebo IG: fish oil capsules (30 mg/kg body weight) Time: 3 months, followed by a 2-month break between treatments.	Among patients on anti-inflammatory diets, the number of tender and swollen joints decreased by 14% during placebo treatment, whereas during fish oil capsules, there were significant reductions in the number of tender (28% vs. 11%) and swollen (34% vs. 22%) joints (*p* < 0.01) and greater EPA enrichment in erythrocyte lipids (244% vs. 217%) and less formation of leukotriene B(4) (34% vs. 8%, *p* > 0.01), 11-dehydro-thromboxane B(2) (15% vs. 10%, *p* < 0.05), and prostaglandin metabolites (21% vs. 16%, *p* < 0.003). A low-AA diet alleviates clinical signs of inflammation in RA patients and potentiates the beneficial effect of fish oil supplementation.
37	Kremer JM et al., 1995 [53]	Clinical Trial	IG: 23 RA patients in mean age 58 years CG: 26 RA patients in mean age 57 years	IG: 130 mg/kg/d omega-3 FAs CG: 9 capsules/d of corn oil Diclofenac placebo was replaced at week 18 or 22, and fish oil supplementation continued for 8 weeks (until week 26 or 30).	In the group taking fish oil, there was a significant reduction in the number of tender joints duration of morning stiffness, global arthritis activity and pain. In patients taking corn oil, none of the clinical parameters improved from baseline. The reduction in the number of tender joints remained significant 8 weeks after discontinuation of diclofenac in patients taking fish oil. IL-1 beta decreased significantly from baseline through weeks 18 and 22 in patients consuming fish oil (−7.7 +/−3.1; *p* = 0.026).
38	Haugen MA et al., 1994 [54]	Clinical Trial	IG: 27 RA patients in mean age 51 years CG: 26 RA patients aged 55 years	IG: 7–10 days of fasting, then a gluten-free vegan diet. For another 3–5 months. After 3.5 months a lacto-vegetarian diet. CG: continuation of normal diet.	Concentrations of 20: 3n-6 and 20: 4n-6 fatty acids were significantly reduced after 3.5 months on a vegan diet (*p* < 0.0001 and *p* < 0.01, respectively), but concentrations increased to baseline values on a lactovegetarian diet. The 20: 5n-3 concentration was significantly reduced after the vegan (*p* < 0.0001) and lactovegetarian (*p* < 0.01) diets. No correlation between changes in fatty acid profiles and clinical improvement.
39	Pullman-Mooar S et al., 1990 [55]	Clinical Trial	7 RA patients in age range of 26–45 years and 7 normal controls	Borage seed oil (9 capsules/d = 1.1 gm/d GLA) Time: 12 weeks	GLA administration increased DGLA ratio, DGLA to AA ratio and DGLA to stearic acid ratio in circulating mononuclear cells. After 12 weeks of GLA supplementation, a significant reduction in the production of PGE2, leukotriene B4 and leukotriene C4 by stimulated monocytes was observed.
40	Jäntti J et al., 1989 [56]	Clinical Trial	I: 10 RA patients in mean age 50 years II: 10 RA patients in mean age 38 years	I: 20 mL evening primrose oil with 9% GLA II: olive oil Time: 12 weeks	Group I showed a decrease in serum levels of oleic acid, EPA, and apolipoprotein B and an increase in serum levels of linoleic acid, GLA, dihomo-gamma-linolenic acid, and AA. Serum EPA concentrations decreased in group II. The decrease in serum EPA and increase in serum AA levels induced by evening primrose oil may not be beneficial in patients with rheumatoid arthritis in light of the role of these FAs as eicosanoid precursors.
41	Cleland LG et al., 1988 [57]	Clinical Trial	RA patients	I: dietary supplementation with fish oil (18 g/d) II: olive oil supplementation Time: 12 weeks	After 12 weeks, the fish oil-treated group showed improvements in tender joint scores and grip strength, a reduction in the mean duration of morning stiffness, a reduction in pain, and a 30% reduction in leukotriene B4 production by isolated neutrophils stimulated in vitro.
42	Belch JJF et al., 1988 [58]	Clinical Trial	I: 16 RA patients aged 48 (30–74) years II: 15 RA patients aged 46 (35–68) years III: 18 RA patients aged 53 (28–73) years	I: 540 mg GLA/day (EPO) II: 240 mg EPA and 450 mg GLA/day (EPO/fish oil), III: oil (placebo). After a 12-month treatment period, a 3-month placebo period was used in all groups.	After 12 months, there was a significant improvement and reduction in NSAID use in the EPO and EPO with fish oil groups. After 3 months of placebo, relapse occurred in those receiving active treatment.
43	Kremer JM et al., 1987 [59]	Clinical Trial	IG: 21 RA patients CG: 19 RA patients	IG: 2.7 g EPA + 1.8 g DHA in 15 MAX-EPA capsules (R.P. Scherer, Clearwater, FL, USA) CG: placebo capsules Timing: 14-week treatment periods and 4-week washout periods.	In the IG group, the mean time to onset of fatigue improved by 156 min and the number of tender joints decreased by 3.5. Production of leukotriene B4 by neutrophils was correlated with a decrease in the number of tender joints (r = 0.53; *p* < 0.05).
44	Beyer K et al., 2021 [60]	Observational study	78 RA patients aged 57.0 ± 12.0 years with varying degrees of periodontitis	No	Elevated phospholipid levels with concomitant decreased choline levels, increased medium-chain acylcarnitines (MC-AC), and decreased MC-AC to long-chain (LC)-AC ratio were associated with prednisolone intake. Higher concentrations of total FA and total cholesterol were found in active RA.
45	Mustonem AM et al., 2019 [61]	Observational study	I: 10 RA patients after total knee replacement II: 10 OA patients after total knee replacement III: 6 atroscopy patients not suffering from RA or OA	No	After treatment, the proportion of omega-6 FAs significantly decreased in the OA and RA groups. The proportion of MUFAs increased in both RA and OA patients. RA patients had a lower proportion of 20: 4n-6, total omega-6 and 22: 6n-3, and a lower omega-3 product/precursor ratio compared with OA patients. Mean FA chain length in synovial fluid decreased in both diagnoses.
46	Nasriati F et al., 2018 [62]	Observational study	35 RA patients with an average age of 45.29 years	No	There was no significant correlation between TNF-α levels and VCAM-1 levels (*p* = 0.677; r = +0.073) or between TNF-α levels and FFA levels (*p* = 0.227; r = −0.21). There was a weak negative correlation of FFA with sVCAM-1.
47	de Pablo P et al., 2018 [63]	Observational study	I: 96 pre-RA subjects II: 258 matched controls	No	The erythrocytic level of omega-6 FA was inversely associated with RA risk, whereas no association was observed with other omega-6 or omega-3 FAs.
48	Beyer K et al., 2018 [64]	Observational study	78 RA patients aged 57 ± 12	No	Patients with omega-3 > 8 index had lower VAS pain severity score and lower periodontal probing depth.
49	Bärebring L et al., 2018 [65]	Observational study	66 RA patients aged 59.9 ± 12.2	No	An omega-3 rich diet with animal fat restriction was not associated with DAS28 (B = −0.02, *p* = 0.787), but high diet quality was significantly negatively associated with hs-CRP (B = −0.6, *p* = 0.044) and ESR (B = −2.4, *p* = 0.002).
50	Gan RW et al., 2017 [66]	Observational study	136 RA patients: I. Anti-CCP2(+) (*n* = 40, aged 43.7 ± 15.4) II. High titre RF(+) (*n* = 27, aged 48.1 ± 13.2) III. RF(−) and anti-CCP2(−) (*n* = 69, aged 46.5 ± 13.9 )	No	Increased omega-3 FA% in RBCs was inversely associated with RF in SE-positive participants and anti-CCP positivity in SE-positive participants, but not in SE-negative participants. In the SERA cohort, use of omega-3 FA supplements was associated with a lower incidence of RF.
51	Jeffery L et al., 2017 [67]	Observational study	22 RA patients aged 53.0 ± 12.5	No	Higher plasma EPA concentrations were associated with greater reduction in DAS28. Plasma EPA PC was positively associated with response to treatment according to EULAR criteria. An increase in Th17 cells after therapy was associated with a lack of response to anti-TNF. ETN increased Th17 frequency in vitro. EPA status was associated with clinical improvement on anti-TNF therapy in vivo and prevented the effects of ETN on Th17 cells in vitro.
52	Gan RW et al., 2016 [68]	Observational study	I: Anti-CCP2 (+) (*n* = 30, aged 45.6 ± 16.5 ) II: Control (Ab−) (*n* = 47, aged 48.6 ± 14.4)	No	The probability of anti-CCP2 was inversely proportional to the total FA omega-3 content in RBCs (0.47; 95% CI 0.24–0.92).
53	Rodríguez-Carrio J et al., 2016 [69]	Observational study	124 RA patients aged 52.47 ± 12.76	No	RA patients showed reduced levels of palmitic (*p* < 0.0001), palmitoleic (*p* = 0.002), oleic (*p* = 0.010), arachidonic (*p* = 0.027), EPA (*p* < 0.0001) and DHA (*p* < 0.0001) acids and an overrepresentation of the NEFA profile compared to HC (*p* = 0.002). FA imbalance may underlie IFNγ production by CD4+ T cells. Changes in NEFA levels were associated with clinical response to TNF-α blockade.
54	Kosinska MK et al., 2015 [70]	Observational study	I: 16 post-mortem donors II: 20 RA patients aged 56 (49–72) III: 26 eOA patients aged 38 (26–56) IV: 22 lOA patients aged 69 (53–74)	No	Significant changes were noted between groups in the relative distribution of PLs and the degree of FA saturation and chain length of FAs. Compared with the control group, more FA-saturated LPC species were reported in the synovial fluid of eOA (63.5% (59.0–70.7%)), lOA (68.8% (65.3–70.6%)) and RA (72.4% (70.2–75.4%)) patients.
55	Di Giuseppe D et al., 2014 [71]	Observational study	32,232 women in whom 205 cases of RA were diagnosed during a 7.5-year follow-up	No	Consumption of omega-3 FAs greater than 0.21 g/day was associated with a 35% reduced risk of developing RA (RR 0.65; 95% CI 0.48–0.90), and consumption of >0.21 g/day was associated with a 52% reduced risk of developing RA. Long-term consumption of ≥1 serving of fish per week compared with <1 serving was associated with a 29% reduced risk (RR 0.71; 95% CI 0.48–1.04).
56	Lee AL. & Park Y, 2013 [72]	Observational study	CG: 100 healthy women aged 50.04 ± 8.00 IG: 100 RA female patients aged 48.39 ± 9.69	no	In RA patients, the levels of ALA, EPA and omega-3 index [EPA + DHA] in erythrocytes were significantly lower than those in the CG. Regression analysis showed that ALA, EPA levels and EPA to AA ratio were negatively associated with RA risk. PGE2 concentration was significantly decreased with increased DHA concentration in erythrocytes of RA patients.
57	Hayashi H et al., 2012 [73]	Observational study	37 RA patients aged 65 ± 9.8 years I: Low disease activity, DAS28 < 3.2 (*n* = 18) II: High disease activity, DAS28 ≥ 3.2 (*n* = 19)	no	Serum leptin and albumin levels were significantly lower, while inflammatory markers were elevated, in the high disease activity group. Dietary assessment showed lower fish oil intake and lower MUFA intake ratio in the high disease activity group. There was a negative correlation between DAS28 and dietary intake in terms of MUFA/FAs intake ratio. Serum oxidative stress marker (reactive oxygen metabolites) showed a positive correlation with DAS28.
58	Ormseth MJ et al., 2011 [74]	Observational study	166 RA patients aged 54.0 (45.0–62.8) and 92 control subjects aged 53.0 (44.8–59.2)	no	Serum FFAs levels were not significantly different in RA patients and controls (0.56 mmol/L (0.38–0.75) and 0.56 mmol/L (0.45–0.70), respectively, *p* = 0.75). In multivariate regression analysis, serum FFAs levels were associated with HOMA-IR (*p* = 0.011), CRP (*p* = 0.01), triglycerides (*p* = 0.005) and Framingham risk score (*p* = 0.048) in RA but not with IL-6 (*p* = 0.48).
59	Elkan AC et al., 2009 [75]	Observational study	80 RA patients in mean age 61.4 ± 12 years	no	A total of 18% of women and 26% of men suffered from rheumatoid cachexia. These patients reported high dietary saturated fat intake, which partially correlated with fatty acid composition in adipose tissue and significantly with disease activity. However, patients with and without cachexia did not differ in their dietary fat intake or in their adherence to the Mediterranean diet.
60	Rosell M et al., 2009 [76]	Observational study	I: 1889 RA patients II: 2145 controls	no	Fatty fish consumption was associated with a moderately reduced risk of developing rheumatoid arthritis (OR 0.8 (95% confidence interval = 0.6–1.0)).
61	Das Gupta AB et al., 2009 [77]	Observational study	I: 50 patients aged 49.9 ± 8.2 years II: 50 patients aged 44.7 ± 7.7 years	I: indomethacin (75 mg/d) II: indomethacin (75 mg/d) and omega-3 FAs (3 g/d) over 12 weeks.	Both groups showed moderate improvement in disease activity after 12 weeks of treatment. Physical functioning, physical role, body pain, general health, vitality, social functioning, grip strength and duration of morning stiffness improved significantly better in the combination group compared with the indomethacin-only treatment group.
62	Pedersen M et al., 2005 [78]	Observational study	57,053 individuals from the Danish National Registry. Sixty-nine individuals developed RA.	no	Increased intake of 30 g/d of fatty fish (≥8 g fat/100 g fish) was associated with a 49% reduction in RA risk (*p* = 0.06), whereas intake of medium-fat fish (3–7 g fat/100 g fish) was associated with a significantly increased RA risk.
63	Gruenwald J et al., 2004 [79]	Observational study	50 RA patients aged between 29 and 73 years	Take 1 capsule of Sanhelios Mussel Lyprinol Lipid Complex (458 mg of fish oil concentrate (50% EPA; 50% DHA) and 35 mg of Lyprinol) twice daily (morning and evening), then from day 3, 2 capsules twice daily. Duration: 12 weeks	A reduction in morning stiffness time, painful and swollen joints was observed at 6 and 12 weeks post-study. Pain was reduced by an average of 60%.
64	Furse RK et al., 2001 [80]	Observational study	healthy volunteers and patients with RA	no	Administration of GLA, an unsaturated fatty acid, reduces joint inflammation in patients with rheumatoid arthritis by inhibiting IL-1 beta release from LPS-stimulated human monocytes. GLA induces a protein that reduces the stability of pro-IL-1 beta mRNA. IL-1 beta is important for host defence, but the enhancement mechanism may be excessive in genetically predisposed patients. Reduction of IL-1 beta autoinduction may therefore be protective in some patients with endotoxic shock and diseases characterised by chronic inflammation.
65	Linos A et al., 1999 [81]	Observational study	145 RA patients and 188 control subjects	no	In multiple regression analysis, consumption of olive oil or cooked vegetables significantly reduced the risk of developing RA (OR: 0.38 and 0.24, respectively).
66	Fraser DA et al., 1999 [82]	Observational study	9 RA patients after completion of 7-day fasting aged 51 (31–65) years	no	Both the concentration of the FFA mixture and the ratio of unsaturated and saturated fatty acids significantly affected lymphocyte proliferation in vitro (*p* < 0.0001).
67	Shapiro JA et al., 1996 [83]	Observational study	324 RA female patients and 1245 controls aged 15–64.	no	Consumption of cooked or baked fish but was associated with a reduced risk of rheumatoid arthritis. Adjusted odds ratios (OR) for 1- < 2 servings and > or =2 servings of cooked or baked fish per week, compared with <1 serving, were 0.78 (95% confidence interval (CI) = 0.53–1.14) and 0.57 (95% CI = 0.35–0.93).
68	Magarò M et al., 1992 [84]	Observational study	20 female RA patients aged between 25 and 45 years	IG: A diet enriched with fish oil (EPA and DHA) CG: current diet	Patients with IG had a significantly lower erythrocyte sedimentation rate and were observed to have improved clinical parameters compared to CG.
69	Jacobsson L et al., 1990 [85]	Observational study	IG1: 21 patients with recently diagnosed RA aged 57 (25–78) years IG2: 21 patients with RA of longer duration (mean 15 years, range 3–43) CG: 32 men and 25 women aged 57 years (range 50–70 years) and had no rheumatic symptoms at the time of the study.	no	The proportion of 18:2 serum phosphatidylcholine correlated inversely with such acute phase proteins as orosomucoid and CRP. It is proposed that decreases in essential FAs are associated with increased desaturase/elongation enzyme activity, increased eicosanoid production, or metabolic changes secondary to a cytokine-mediated inflammatory response. However, ascorbic acid levels were lower in RA and correlated inversely with haptoglobin, orosomucoid, and CRP levels, indicating an association between ascorbic acid levels and degree of inflammation.
70	Sperling RI et al., 1987 [86]	Observational study	12 RA patients	20 g/d of Max-EPA fish oil for 6 weeks	After fish oil supplementation, the AA:EPA ratio in neutrophil cell lipids decreased from 81:1 to 2.7:1, and mean leukotriene B4 production decreased by 33%. There was also a 37% decrease in platelet-activating factor production at week 6. Fish oil supplementation may have anti-inflammatory effects.
71	Klickstein LB et al., 1980 [87]	Observational study	Synovial fluid and synovial tissue sonication of patients with RA, SA and NIA	no	The concentration of 5(S),12(R)-dihydroxy-6,8,10-(trans/trans/cis)-14-cis-eicosatetraenoic acid (leukotriene B4) in synovial fluid was significantly elevated in patients with RA and rheumatoid factor present (*p* < 0.05, *n* = 14) and in patients with SA (*p* < 0.05, *n* = 10), compared with those with NIA (*n* = 9). The content of 5(S)-hydroxy-6,8,11,14-eicosatetraenoic acid (5-HETE), but not leukotriene B4, was significantly elevated in the synovial tissue of seven RA patients compared with four NIA subjects (*p* < 0.05). A single intra-articular corticosteroid injection significantly decreased leukotriene B4 levels in synovial fluid of six RA patients. These data suggest the involvement of the potent chemotactic factors 5-HETE and leukotriene B4 in human inflammatory disease.

RA: Rheumatoid arthritis; DMARDs: disease modifying anti-rheumatic drugs; DAS 28: Disease Activity Score of 28 joints; VAS: Visual Analogue Scale; hs-CRP: high sensitivity-C-Reactive Protein; PUFA: polyunsaturated fatty acids; anti-CCP: Anti-cyclic citrullinated peptide; RF: Rheumatoid Factor; PGE2: prostaglandin E2; TNF: tumour necrosis factor; IL: interleukin; EPA: eicosapentaenoic acid; DHA: docosahexaenoic acid; RCT: randomised controlled trial; AA: arachidonic acid; LA: linoleic acid; FA: Fatty Acid; OA: osteoarthritis; ACR: American College of Rheumatology; RBC: red blood cell; The NEFA: non-esterified fatty acid; SE: shared epitope; SERA: The Scottish Early Rheumatoid Arthritis; ETN: etanercept; IG: intervention group; CG: control group; CI: confidential intervals; RR: risk ratio; HMLE: hard-shelled mussel; MUFA: Monounsaturated Fatty Acid; VCAM-1: vascular cell adhesion molecule 1; FFA: free fatty acids; EULAR: European Alliance of Associations for Rheumatology; mHAQ: Health Assessment Questionnaire; PLs: phospholipid species; eOA: early osteoarthritis; lOA: late osteoarthritis; IFN: interferon; PC: plasma phosphatidylcholine; MC-AC: medium-chain acylcarnitines; LPC: lysophosphatidylcholine; EL: erythrocyte lipids; NMR: Nuclear Magnetic Resonance; EPO: evening primrose oil; GLA: gamma-linolenic acid; CLAs: conjugated linoleic acids; HOMA-IR: homeostasis model assessment of insulin resistance; LDL-C: low-density lipoprotein cholesterol; HDL-C: high-density lipoprotein cholesterol; SA: spondyloarthropathy; NIA: noninflammatory arthropathy.

## Data Availability

The data that support the findings of this study are available from the corresponding author upon reasonable request.

## Abbreviations

RA: Rheumatoid arthritis; DMARDs: disease modifying anti-rheumatic drugs; DAS 28: Disease Activity Score of 28 joints; VAS: Visual Analogue Scale; SF-36: Short Form Health survey; hs-CRP: high sensitivity C-Reactive Protein; PUFA: polyunsaturated fatty acids; anti-CCP: Anti-cyclic citrullinated peptide; RF: Rheumatoid Factor; PGE2: prostaglandin E2; TNF: tumour necrosis factor; IL: interleukin; EPA: eicosapentaenoic acid; DHA: docosahexaenoic acid; PRISMA: the preferred reporting items for systematic reviews and meta-analyses; RCT: randomised controlled trial; CASP: critical appraisal skills program; AA: arachidonic acid; LC-PUFA: Long-Chain Polyunsaturated Fatty Acids; ALA: alpha-linolenic acid; LA: linoleic acid; FA: Fatty Acid; CDAI: clinical disease activity index; OA: osteoarthritis; ROM: range of motion; ACR: American College of Rheumatology; RBC: red blood cell; HLA: Human Leukocyte Antigen; NEFA: non-esterified fatty acid; SE: shared epitope; SERA: Scottish Early Rheumatoid Arthritis; ETN: etanercept; ESR: erythrocyte sedimentation rate; SLE: systemic lupus erythematous; IG: intervention group; CG: control group; CI: confidential intervals; RR: risk ratio; HMLE: hard-shelled mussel; MUFA: Monounsaturated Fatty Acid; VCAM-1: vascular cell adhesion molecule 1; FFA: free fatty acids; EULAR: European Alliance of Associations for Rheumatology; mHAQ: Health Assessment Questionnaire; PLs: phospholipid species; eOA: early osteoarthritis; lOA: late osteoarthritis; IFN: interferon; PC: plasma phosphatidylcholine; MC-AC: medium-chain acylcarnitines; LPC: Lysophosphatidylcholine; EL: erythrocyte lipids; NMR: Nuclear Magnetic Resonance; EPO: evening primrose oil; GLA: gamma-linolenic acid; CLAs: conjugated linleic acids; HOMA-IR: homeostasis model assessment of insulin resistance; LDL-C: low-density lipoprotein cholesterol; HDL-C: high-density lipoprotein cholesterol; SA: spondyloarthropathy; NIA: noninflammatory arthropathy.
